# Determining utility values in patients with anterior cruciate ligament tears using clinical scoring systems

**DOI:** 10.1186/1472-6963-11-182

**Published:** 2011-08-04

**Authors:** Mazda Farshad, Christian Gerber, Thomas Szucs, Dominik C Meyer

**Affiliations:** 1 Balgrist University Hospital, Orthopedic Surgery, University of Zürich, Switzerland; 2 Institute of Pharmaceutical Medicine, University of Basel, Switzerland

## Abstract

**Background:**

Several instruments and clinical scoring systems have been established to evaluate patients with ligamentous knee injuries. A comparison of individual articles in the literature is challenging, not only because of heterogeneity in methodology, but also due to the variety of the scoring systems used to document clinical outcomes. There is limited information about the correlation between used scores and quality of life with no information being available on the impact of each score on the utility values. The aim of this study was to compare the most commonly used scores for evaluating patients with anterior cruciate ligament (ACL) injuries, and to establish corresponding utility values. These values will be used for the interpretation and comparison of outcome results in the currently available literature for different treatment options.

**Methods:**

Four hypothetical vignettes were defined, based on different levels of activities after rupture of the ACL to simulate typical situations seen in daily practice. A questionnaire, including the Health Utility Index (HUI) for utility values, the IKDC subjective score, the Lysholm and the Tegner score, was created and 25 orthopedic surgeons were asked to fill the questionnaire for each hypothetical patient as proxies for all patients they had treated and who would fit in that hypothetical vignette.

**Results:**

The utility value as an indicator for quality of life increased with the level of activity. Having discomforts already during normal activities of daily living was rated with a mean utility value of 0.37 ± 0.19, half of that of a situation where mild sport activity was possible without discomfort (0.78 ± 0.11). All investigated scores were able to distinguish clearly (p < 0.05) between the hypothetical vignettes. However, the utility values correlated best with the IKDC subjective score (r = 0.86, p < 0.001) followed by the Lysholm score (r = 0.77, p < 0.001) and the Tegner score (r = 0.77, p < 0.001).

**Conclusions:**

Here we report the correlation between the most commonly used scores for the assessment of patients with a ruptured ACL and utility values as an indicator of quality of life. Assumptions were based on expert opinions to provide a possible transformation algorithm. The IKDC subjective knee score showed the highest correlation to the quality of life (i.e. HUI) in patients with a ruptured ACL. Confirmation of our results is needed by systematic inclusion of a measurement instrument for utility values in future clinical studies beside the already used clinical knee scoring systems.

## Background

Rupture of the anterior cruciate ligament (ACL) changes the kinematics of the knee [1] and often results in instability with accompanying functional disability and pain [2-20]. There are more than thousands of scientific articles [21] illuminating several aspects on the ACL, however there is no absolute consensus on the optimal treatment. While some reported reasonable outcomes after the operative treatment with various techniques [2-12], others documented sufficient clinical results after conservative treatment [13-20]. A decision towards one or the other treatment option is challenging and should include assessment of individual factors, such as time since injury, patients level of activity, amount of instability episodes despite activity modifications, patients compliance for postoperative rehabilitation programs or performance of physiotherapy as a corner stone of the conservative approach. However, beside individual factors, the decision should also consider economic and public health aspects. Non-technical factors such as the quality of life might bring new arguments toward one or the other option. Several instruments and scoring systems [22-26] have been used to assess and evaluate patients with injuries to knee ligaments. Comparing the individual articles is challenging, not only as a result of heterogeneities in investigated populations, treatment protocols and techniques, but also because of the use of the variety of scoring systems. There is limited information about the correlation of the used scores to the quality of life [24,27-30] and, to our knowledge, no information on impact of each score to the utility values. Utilities, as understood in health economics, are values that reflect an individual's preferences for different health outcomes measured on a scale with one reflecting a state of perfect health and zero being the state of death [31]. The scores are used to generate quality-adjusted life years (QALY's) for use of cost-utility analysis.

The aim of this survey study was to correlate the most commonly used scores for the evaluation of patients with ACL injury, namely the IKDC score, the Lysholm score and the Tegner score [22], not only between each other but also to the quality of life, to verify the ability of the scores to discriminate among different situations and to provide information for the transformation of reported outcomes to utility values. The hypothesis is that there is a correlation between commonly used scores for assessment of patients with a ruptured ACL and utility values as an indicator of quality of life.

## Methods

A survey including the most commonly used scoring systems for evaluation and reporting outcomes of different treatments of anterior cruciate ligament (ACL) ruptures, namely the IKDC score, the Lysholm score and the Tegner score [22]was created. The IKDC score is a knee specific, however, not disease specific score and consists of a demographic form, current health assessment form, subjective knee evaluation form, knee history form, surgical documentation form, and knee examination form [32]. Researchers preferably use the subjective knee evaluation forms and knee examination forms. The subjective evaluation part consists of 10 questions to be answered by the patient. The Lysholm score was designed with emphasis on follow up for symptoms of instability after knee ligament surgery [23] and consists of 5 symptoms and 3 types of activities with each different levels of intensities to be rated by the patient. The Tegner score focuses on activity after a knee ligament lesion [26] based on 10 activity levels.

The Health Utility Index (HUI)[33] was added to the questionnaire to assess utility values. Four hypothetical vignettes (Table 1) of patients with a unilateral isolated rupture of their anterior cruciate ligament and who had varying degrees of activity levels were presented to 25 orthopedic surgeons of the same institution. The hypothetical vignettes were constructed, based on levels of activities of patients after an ACL rupture as introduced and validated by Gottlob et al [34] with the aim to represent the whole range of patients seen in daily practice. Limited information was given for each hypothetical vignette to narrow the range of patients that would fit for each vignette and particularly no information was given regarding the treatment choice of the patients. The cross sectional design of the study asked for completion of the questionnaires for each hypothetical patient by the surgeons. This served as a proxy for the average patient that they would assess in practice which would be similar to the hypothetical patient in the presented vignette. The activity levels 0 (death) and V (perfect health) were defined as minimum and maximum, respectively, in each scoring system. Based on the results of the questionnaire, scores and the health utility value could be constructed for each of the hypothetical patients. For calculation of the utility values, the answer of each expert on behalf of the hypothetical patients on the Health Utility Index was used as described by Torrance et al [35]. Statistical analysis was performed with the software PRISM (Version 4, Graphpad software, La Jolla (CA), USA) and SPSS (Version 19.0 for Macintosh). Grouped internal data was tested for normal distribution using the Kolmogorov Smirnov test before using either Pearson correlation for Gaussian population distribution or Spearman correlation for nonparametric data, respectively. A repeated measure ANOVA analysis with Greenhouse-Geisser correction was performed to test the ability of the scores to distinguish between the constructed vignettes. Level of significance was set at p < 0.05.

**Table 1:** Characteristics of the hypothetical vignettes

Vignette	Hypothetical History of the Patient	Level of Activity
1	35 year old patient with symptomatic ADLs	I
2	35 year old patient who can perform ADLs without symptoms but no kind of sports	II
3	35 year old patient with knee symptoms with mildly stressful sports (e.g. jogging, swimming, biking)	III
4	35 year old patient with knee symptoms with moderately stressful sports (e.g. baseball, alpine skiing, dance)	IV

Hypothetical vignettes were based on activity levels I to IV as described by Gottlob et al [34].

## Results

### Utility values

**Figure 1:**
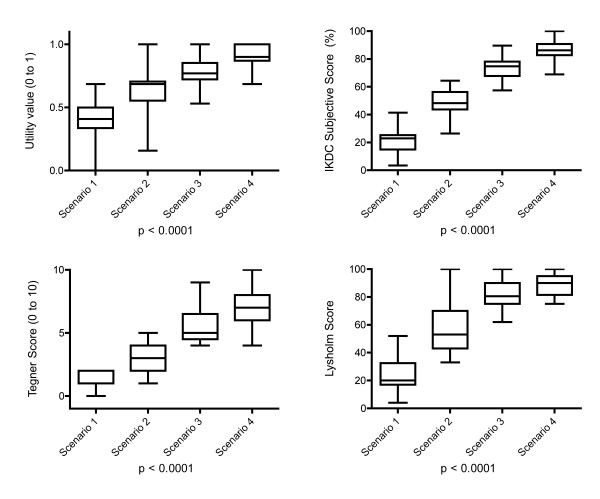
**Hypothetical scenarios and scores**. The IKDC subjective knee score, Lysholm and Tegner score for different activity levels (I to IV for hypothetical vignettes (1 to 4)).

The utility value, as an indicator for quality of life, increased with the level of activity (Figure 1). Having problems with the knee already during usual activities of daily living was rated with a mean utility value of 0.37 ± 0.19, whereas in those vignettes, where daily activities could be performed without discomfort, the utility values were significantly higher, even more than double if some kind of sport could be performed without discomfort as in vignettes 3 and 4. The difference in gain of utility values from vignettes 1 to 2 (0.26 ± 0.15) was significantly higher than from vignettes 3 to 4 (0.13 ± 0.12).

### Knee scores

All used scores were able to distinguish clearly (p < 0.05) between the hypothetical vignettes (Figure 1 and Table 2). However, the differences between values in vignettes 1 and 2 were more pronounced than those between the vignettes 2 and 3 or 3 and 4 (Table 2), respectively. The difference of values for a hypothetical patients without discomfort during activities of daily live and who have discomfort with any kind of sport activities (vignette 2) compared to those who do not have any discomfort with mild sport activities (vignette 3) is less than the difference of those patients who have (vignette 1) or have not (vignette 2) discomfort during usual activity of daily living (Table 3) in the Lysholm score, nearly equal in the IKDC subjective score and higher in the Tegner score. However, this did not correspond to the differences observed in the utility values, which were influenced the most by discomfort of the knee during usual activities of daily living (Table 3). The strongest correlations between the evaluated knee scores were found between the IKDC subjective score and the Lysholm score (r = 0.92, p < 0.05) followed by Tegner score (r = 0.89, p < 0.05). These correlations were still significant if each vignette was analyzed separately other than in vignette 3 for the correlation of the IKDC and the Tegner score (r = 0.08, p = 0.67).

**Table 2:** Utility values, IKDC, Lysholm and Tegner scores for each of the activity levels (0-V)

Level of Activity	0	I	II	III	IV	V
		**mean ± ***SD*	**mean ± ***SD*	**mean ± ***SD*	**mean ± ***SD*	
**Utility value (HUI)**	**0**	**0.37 ± ***0.19*	**0.63 ± ***0.16*	**0.78 ± ***0.11*	**0.91 ± ***0.09*	**1**
**IKDC subjective score**	0	22 **± ***9*	48 **± ***10*	73 **± ***8*	86 **± ***8*	100
**Lysholm score**	0	25 **± ***12*	57 **± ***17*	80 **± ***10*	89 **± ***8*	100
**Tegner score**	0	1.2 **± ***0.7*	2.9 **± ***0.9*	5.6 **± ***1.5*	7.2 **± ***1.7*	10.0

The hypothetical vignettes could be clearly distinguished by all score systems.

**Table 3:** Differences in Utility values, IKDC, Lysholm and Tegner scores for each of the Activity levels

Differences	II-I	III-II	IV-III
	**mean ± **SD	**mean ± **SD	**mean ± **SD
**Utility value (HUI)**	0.26 **± **0.15	0.15 **± **0.13	0.13 **± **0.12
**IKDC subjective score**	26 **± **9	25 **± **8	13 **± **7
**Lysholm score**	33 **± **11	22 **± **12	9 **± **9
**Tegner score**	1.6 **± **1.1	2.5 **± **1.6	1.5 **± **1.2

The difference in gain of utility value from vignettes 1 to 2 (0.26 ± 0.15) was significantly higher than from vignettes 3 to 4 (0.13 ± 0.12).

### Correlations of knee scores to utility values

**Figure 2:**
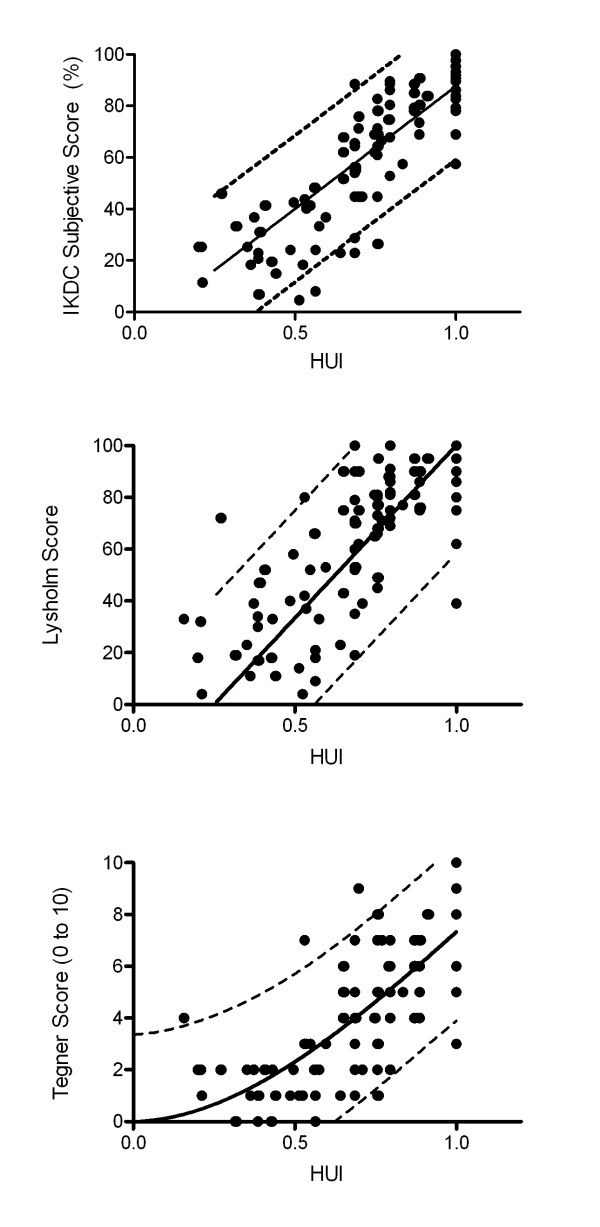
**The correlation of ACL scores to the utility values**. All scores correlated significantly with the utility values (p < 0.05), however, the correlation was strongest for the IKDC subjective knee score (r = 0.86, 95% CI: 0.81, 0.90). Either linear regression (A and B) or third order polynomial non-linear regression (C) was used to find a curve representing most reasonably the trend of the correlations.

Overall, the utility values of all vignettes correlated best with the IKDC subjective score (r = 0.86, p < 0.001) followed by the Lysholm score (r = 0.77, p < 0.001) and the Tegner score (r = 0.77, p < 0.001) (Figure 2). However, for each vignette analyzed separately, the correlation of IKDC to the utility values was weaker (vignette 2, r = 0.36, p < 0.05; vignette 3, r = 0.54, p < 0.05; vignette 4, r = 0.39, p < 0.05) and there was no correlation in vignette 1 (r = -0.24, p = 0.2).

## Discussion

The purpose of this study was to investigate the correlation between the most commonly used scores for assessment of patients with an injury to the ACL and the utility values, as an indicator for the quality of life for potential transformation of currently available evidence on clinical outcome of differently treated patients. Further, the ability of the scores to discriminate unequal situations was assessed. We found that each of the scores, namely the IKDC subjective score, Lysholm score and the Tegner score were able to distinguish between the hypothetical patients (Figure 1). Further, a strong correlation was found between the IKDC subjective score and the utility values (Figure 2), followed by the Lysholm and the Tegner score. We were able to provide a potential key for transformation of the IKDC, Lysholm and Tegner score to utility values as an indicator of the quality of life (Table 1, Figure 1 and 2). The correlations found here could be used to transform reported score-values of IKDC subjective, Lysholm and Tegner score to utility values based on the Figure 2. IKDC subjective score has been designed for patients with a wide range of knee problems and was not designed to specifically address only patients with torn ACL. It however, was most strongly correlated with utility values. Although both the Lysholm and Tegner score were initiated first for patients with knee ligament injuries, they did not perform better than the IKDC subjective score in the setting of our study.

We constructed the hypothetical vignettes based on the levels of activities as described by Gottlob et al [34]. A change of a very low level of activity to a higher level (I to II) gained more on the utility value and seems more worth subjectively than change from an already good level of activity to a high level of activity (III to IV). The utility values which were found here for each vignette and activity level are similar in tendency but are, on average, higher than those described by Gottlob et al [34] for each of the individual activity levels. This difference could be explained by the difference in the setting; by time of analysis (> 10 years difference) and location (USA and Europe). Further, the utility values reported by Gottlob et al [34] have been generated by young healthy students not exposed to ACL patients on a non-validated measurement system for utility values. We feel that the cohort used by Gottlob et al might have overestimated the impact of ACL injuries on the utility values and it seems reasonable to prefer experts to assess how hypothetical patients would perform on a validated health utility questionnaire.

Our results can be used for the transformation of the currently available outcome data (scores) of different treatment options for ACL patient to the utility values. This might be used not only by health professionals interested in the impact of different treatment options on the quality of life but also and for cost utility analyses. However, our results must be interpreted with caution to the limitations of the study: First, the results are based on opinions of physicians of the same institution on how the hypothetical patient in average would perform and not on real patient data. Although the approach of assuming values based on expert's opinion is frequently used in health economic research [36-38], it might bear potential disadvantages; two experts might not agree fully on their opinions. We tried to reduce the potential of discrepancy of opinions by including a large number of experts. Further, experts might not represent accurately their patients as surrogates. However, we believe that for production of a transformation key from knee scores to the utility values, such as performed here, using real patient data might bear too much uncontrollable confounders and that standardized hypothetic patients might better represent the cohort of all ACL patients. Second, the designed hypothetical patients might not perfectly represent the range of patients seen in daily practice with a variety of potential confounders such as age, gender, comorbidities and activity level. However, we based the construction of the vignettes on the activity scoring system of Gottlob et al [34] and found that the constructed patients are very well distributed and cover the possible ranges of all scoring systems (Figure 1). We used an average age of 35 years for the clinical vignettes based on the Swiss national statistics and our own unpublished data of patients with ACL injury. The influence of age as a patient characteristic is however difficult and complex to grasp and might be reflected in many other, in part competing aspects: Younger age may simultaneously mean a tendency towards higher activity, higher ability to compensate for a ligamentous deficit, better healing, less weight, higher expectations, a more physical strenuous profession, etc. However, it was not the aim of this study to investigate the potential effect of age on utility values and the current standardization to a defined type of patient should unlikely influence the comparison of the different scoring systems.

## Conclusions

Here we report the correlative strength of relations between the most commonly used scores for the assessment of patients with a ruptured ACL and utility values as a measure of quality of life. This information can be used to transform available outcome scores, as reported currently by knee scoring systems, to utility values as needed for a cost effectiveness analysis. Assumptions were based on clinical vignettes and expert opinions and provide a potential transformation key. The IKDC subjective knee score behaved as the most suitable surrogate for evaluation of quality of life in patients with a ruptured ACL in this study. Inclusion of a measurement instrument for utility values in future prospective clinical studies, in addition to commonly used knee scoring systems, would be ideal to confirm the results of this present study.

## Competing interests

The authors declare that they have no competing interests.

## Authors' contributions

All authors have made substantial contributions to this study; MF was involved in conception and design and the acquisition of data, analysis and interpretation of data and in drafting the manuscript. DCM was involved in conception and design and the acquisition of data, interpretation of data and revising the manuscript critically for important intellectual content. TS was involved in conception and design and interpretation of data and revising the manuscript critically for important intellectual content. GC was involved in conception and design and the acquisition and interpretation of data, revising the manuscript critically for important intellectual content and supervision and coordination of the research group. All authors read and approved the final manuscript.

## Pre-publication history

The pre-publication history for this paper can be accessed here:

http://www.biomedcentral.com/1472-6963/11/182/prepub

## References

[B1] Van de Velde SK Gill TJ Li G Evaluation of kinematics of anterior cruciate ligament-deficient knees with use of advanced imaging techniques, three-dimensional modeling techniques, and robotics J Bone Joint Surg Am 2009 91 Suppl 1 108 114 19182035 10.2106/JBJS.H.01382 PMC2663348

[B2] Aglietti P Buzzi R D'Andria S Zaccherotti G Long-term study of anterior cruciate ligament reconstruction for chronic instability using the central one-third patellar tendon and a lateral extraarticular tenodesis Am J Sports Med 1992 20 1 38 45 10.1177/036354659202000111 1554072

[B3] Almqvist KF Willaert P De Brabandere S Criel K Verdonk R A long-term study of anterior cruciate ligament allograft reconstruction Knee Surg Sports Traumatol Arthrosc 2009 17 7 818 822 10.1007/s00167-009-0808-y 19421736

[B4] Fink C Hoser C Hackl W Navarro RA Benedetto KP Long-term outcome of operative or nonoperative treatment of anterior cruciate ligament rupture--is sports activity a determining variable? Int J Sports Med 2001 22 4 304 309 10.1055/s-2001-13823 11414676

[B5] Harner CD Olson E Irrgang JJ Silverstein S Fu FH Silbey M Allograft versus autograft anterior cruciate ligament reconstruction: 3- to 5-year outcome Clin Orthop Relat Res 1996 324 134 144 10.1097/00003086-199603000-00016 8595749

[B6] Hertel P Behrend H Cierpinski T Musahl V Widjaja G ACL reconstruction using bone-patellar tendon-bone press-fit fixation: 10-year clinical results Knee Surg Sports Traumatol Arthrosc 2005 13 4 248 255 10.1007/s00167-004-0606-5 15690197

[B7] Kessler MA Behrend H Henz S Stutz G Rukavina A Kuster MS Function, osteoarthritis and activity after ACL-rupture: 11 years follow-up results of conservative versus reconstructive treatment Knee Surg Sports Traumatol Arthrosc 2008 16 5 442 448 10.1007/s00167-008-0498-x 18292988

[B8] Krych AJ Jackson JD Hoskin TL Dahm DL A meta-analysis of patellar tendon autograft versus patellar tendon allograft in anterior cruciate ligament reconstruction Arthroscopy 2008 24 3 292 298 10.1016/j.arthro.2007.08.029 18308180

[B9] Liden M Ejerhed L Sernert N Laxdal G Kartus J Patellar tendon or semitendinosus tendon autografts for anterior cruciate ligament reconstruction: a prospective, randomized study with a 7-Year follow-up Am J Sports Med 2007 35 5 740 748 10.1177/0363546506298275 17293471

[B10] Ruiz AL Kelly M Nutton RW Arthroscopic ACL reconstruction: a 5-9 year follow-up Knee 2002 9 3 197 200 10.1016/S0968-0160(02)00019-4 12126677

[B11] Strand T Molster A Hordvik M Krukhaug Y Long-term follow-up after primary repair of the anterior cruciate ligament: clinical and radiological evaluation 15-23 years postoperatively Arch Orthop Trauma Surg 2005 125 4 217 221 10.1007/s00402-004-0766-2 15875231

[B12] Taylor DC Posner M Curl WW Feagin JA Isolated tears of the anterior cruciate ligament: over 30-year follow-up of patients treated with arthrotomy and primary repair Am J Sports Med 2009 37 1 65 71 19029313 10.1177/0363546508325660

[B13] Ahn JH Chang MJ Lee YS Koh KH Park YS Eun SS Non-operative treatment of ACL rupture with mild instability Arch Orthop Trauma Surg10.1007/s00402-010-1077-4 20336305

[B14] Barrack RL Bruckner JD Kneisl J Inman WS Alexander AH The outcome of nonoperatively treated complete tears of the anterior cruciate ligament in active young adults Clin Orthop Relat Res 1990 259 192 199 2208856

[B15] Daniel DM Stone ML Dobson BE Fithian DC Rossman DJ Kaufman KR Fate of the ACL-injured patient. A prospective outcome study Am J Sports Med 1994 22 5 632 644 10.1177/036354659402200511 7810787

[B16] Goldstein J Bosco JA The ACL-deficient knee: natural history and treatment options Bull Hosp Jt Dis 2001 60 3-4 173 178 12102406

[B17] Kostogiannis I Ageberg E Neuman P Dahlberg L Friden T Roos H Activity level and subjective knee function 15 years after anterior cruciate ligament injury: a prospective, longitudinal study of nonreconstructed patients Am J Sports Med 2007 35 7 1135 1143 10.1177/0363546507299238 17351121

[B18] McDaniel WJ Jr Dameron TB Jr The untreated anterior cruciate ligament rupture Clin Orthop Relat Res 1983 172 158 163 6821986

[B19] Noyes FR Mooar PA Matthews DS Butler DL The symptomatic anterior cruciate-deficient knee. Part I: the long-term functional disability in athletically active individuals J Bone Joint Surg Am 1983 65 2 154 162 6687391 10.2106/00004623-198365020-00003

[B20] Strehl A Eggli S The value of conservative treatment in ruptures of the anterior cruciate ligament (ACL) J Trauma 2007 62 5 1159 1162 10.1097/TA.0b013e31805006e7 17495718

[B21] Frank CB Jackson DW The science of reconstruction of the anterior cruciate ligament J Bone Joint Surg Am 1997 79 10 1556 1576 9378743 10.2106/00004623-199710000-00014

[B22] Johnson DS Smith RB Outcome measurement in the ACL deficient knee--what's the score? Knee 2001 8 1 51 57 10.1016/S0968-0160(01)00068-0 11248569

[B23] Lysholm J Gillquist J Evaluation of knee ligament surgery results with special emphasis on use of a scoring scale Am J Sports Med 1982 10 3 150 154 10.1177/036354658201000306 6896798

[B24] Mohtadi N Development and validation of the quality of life outcome measure (questionnaire) for chronic anterior cruciate ligament deficiency Am J Sports Med 1998 26 3 350 359 9617395 10.1177/03635465980260030201

[B25] Noyes FR McGinniss GH Grood ES The variable functional disability of the anterior cruciate ligament-deficient knee Orthop Clin North Am 1985 16 1 47 67 3881716

[B26] Tegner Y Lysholm J Rating systems in the evaluation of knee ligament injuries Clin Orthop Relat Res 1985 198 43 49 4028566

[B27] Calvisi V De Vincentiis B Palumbo P Padua R Lupparelli S Health-related quality of life in patients with anterior cruciate ligament insufficiency undergoing arthroscopic reconstruction: a practice-based Italian normative group in comorbid-free patients J Orthop Traumatol 2008 9 4 233 238 10.1007/s10195-008-0034-2 19384492 PMC2657332

[B28] Hartwick M Meeuwisse W Vandertuin J Maitland M Knee pain in the ACL-deficient osteoarthritic knee and its relationship to quality of life Physiother Res Int 2003 8 2 83 92 10.1002/pri.275 12879730

[B29] Moller E Weidenhielm L Werner S Outcome and knee-related quality of life after anterior cruciate ligament reconstruction: a long-term follow-up Knee Surg Sports Traumatol Arthrosc 2009 17 7 786 794 10.1007/s00167-009-0788-y 19360401

[B30] Ochiai S Hagino T Tonotsuka H Haro H Health-related quality of life in patients with an anterior cruciate ligament injury Arch Orthop Trauma Surg 130 3 397 399 10.1007/s00402-009-0964-z 19756671

[B31] Torrance GW Measurement of health state utilities for economic appraisal J Health Econ 1986 5 1 1 30 10.1016/0167-6296(86)90020-2 10311607

[B32] Irrgang JJ Anderson AF Boland AL Harner CD Kurosaka M Neyret P Richmond JC Shelborne KD Development and validation of the international knee documentation committee subjective knee form Am J Sports Med 2001 29 5 600 613 11573919 10.1177/03635465010290051301

[B33] Horsman J Furlong W Feeny D Torrance G The Health Utilities Index (HUI): concepts, measurement properties and applications Health Qual Life Outcomes 2003 1 54 10.1186/1477-7525-1-54 14613568 PMC293474

[B34] Gottlob CA Baker CL Jr Pellissier JM Colvin L Cost effectiveness of anterior cruciate ligament reconstruction in young adults Clin Orthop Relat Res 1999 367 272 282 10546625

[B35] Torrance GW Utility approach to measuring health-related quality of life J Chronic Dis 1987 40 6 593 603 10.1016/0021-9681(87)90019-1 3298297

[B36] Gold M Siegel J Russell L Weinstein M Cost-effectiveness in health and medicine 1996 New York: Oxford University Press

[B37] Griffin SC Weatherly HL Richardson GA Drummond MF Methodological issues in undertaking independent cost-effectiveness analysis for NICE: the case of therapies for ADHD Eur J Health Econ 2008 9 2 137 145 10.1007/s10198-007-0052-7 17476538

[B38] Drummond MF Jefferson TO Guidelines for authors and peer reviewers of economic submissions to the BMJ. The BMJ Economic Evaluation Working Party BMJ 1996 313 7052 275 283 8704542 10.1136/bmj.313.7052.275 PMC2351717

